# Long non-coding RNA LINC00488 facilitates thyroid cancer cell progression through miR-376a-3p/PON2

**DOI:** 10.1042/BSR20201603

**Published:** 2021-03-02

**Authors:** Fuyuan Xie, Longgen Li, Yuting Luo, Rensheng Chen, Jinhong Mei

**Affiliations:** 1 Department of Pathology, The First Affiliated Hospital of NanChang University, Nanchang, China; 2 Department of General Thyroid Surgery, The First Affiliated Hospital of NanChang University, Nanchang, China

**Keywords:** Apoptosis, LncRNA LINC00488, miR-376a-3p, PON2, Proliferation, Thyroid cancer

## Abstract

**Objective:** Long non-coding RNAs (lncRNAs) recently have been identified as influential indicators in a variety of malignancies. The aim of the present study was to identify a functional lncRNA LINC00488 and its effects on thyroid cancer in the view of cell proliferation and apoptosis.

**Methods:** In order to evaluate the effects of LINC00488 on the cellular process of thyroid cancer, we performed a series of *in vitro* experiments, including cell counting kit-8 (CCK-8) assay, EdU (5-ethynyl-2′-deoxyuridine) assay, flow cytometry, transwell chamber assay, Western blot and RT-qPCR. The target gene of LINC00488 was then identified by bioinformatics analysis (DIANA and TargetScan). Finally, a series of rescue experiments was conducted to validate the effect of LINC00488 and its target genes on proliferation, migration, invasion and apoptosis of thyroid cancer.

**Results:** Our findings revealed that LINC00488 was highly expressed in thyroid cancer cell lines (BCPAP, BHP5-16, TPC-1 and CGTH-W3) and promoted the proliferation, migration and invasion, while inhibited the apoptosis of thyroid cancer cells (BCPAP and TPC-1). The results of bioinformatics analysis and dual luciferase reporter gene assay showed that LINC00488 could directly bind to miR-376a-3p and down-regulated the expression level of miR-376a-3p. In addition, Paraoxonase-2 (PON2) was a target gene of miR-376a-3p and negatively regulated by miR-376a-3p. Rescue experiment indicated that LINC00488 might enhance PON2 expression by sponging miR-376a-3p in thyroid cancer.

**Conclusion:** Taken together, our study revealed that lncRNA LINC00488 acted as an oncogenic gene in the progression of thyroid cancer via regulating miR-376a-3p/PON2 axis, which indicated that LINC00488-miR-376a-3p-PON2 axis could serve as novel biomarkers or potential targets for the treatment of thyroid cancer.

## Introduction

Thyroid carcinoma has become the most frequently occurring endocrine malignancy and its incidence is annually increasing in the world [1,2]. According to data from 2018 National Cancer Institute, thyroid carcinoma ranks ninth in tumor incidence. The incidence of thyroid carcinoma is approximately three times in women than that in men [3]. Most of the thyroid cancer cases originated from parafollicular or follicular thyroid cells, which were divided into papillary thyroid cancer (PTC), follicular thyroid cancer (FTC) and anaplastic thyroid cancer (ATC) according to the pathological types [4,5]. At present, chemotherapy and surgery have become the mainstay treatment modalities for thyroid cancer patients [6,7]. However, drug resistance, undesirable side effects of chemotherapy as well as the postoperative recurrence remain the major problem in the treatment of thyroid cancer [8]. Therefore, it is an urgent need for precise clinical diagnosis at the early stage of thyroid cancer. The molecular markers may prove to be useful tools regarding the appropriate understanding of molecular mechanisms of thyroid cancer [9,10]. In recent years, more and more evidence have revealed that the function of non-coding genes especially long non-coding RNAs (lncRNAs) display a distinct ability to regulate protein-coding and non-coding genes at transcriptional or post-transcriptional level, which act as a set of new regulators that participate in various cellular functions and disease processes [11,12]. Hence, it is of great interest to provide a fresh insight for investigation into the potential lncRNAs associated with thyroid cancer.

LncRNAs, a kind of largely functional transcript above 200 nucleotides in length, possess multiple biological functions, including the regulation of cell cycle and cellular differentiation via transcription, translation, epigenetic modification of target genes [13]. Accumulating studies have reported that lncRNAs are related with the development, progression and metastasis of various types of cancer [14–16]. At present, some lncRNAs have been reported to be expressed abnormally in thyroid cancer. For example, lncRNA FOXD2-AS1 could serve as a novel recurrent marker or a potential target in thyroid cancer [17] and lncRNA ZFAS1 could serve as a novel potential biomarker for predicting the prognosis of thyroid cancer [18]. LncRNA LINC00488 has been reported overexpressed in various types of cancer, indicating that lncRNA LINC00488 may participate in the progression of these cancers [12,19]. Nevertheless, to date, the functional role of LINC00488 in the progression of thyroid cancer is unclear.

In the present study, the aim was to reveal the key functions of LINC00488 on the proliferation, apoptosis, migration and invasion of thyroid cancer cells *in vitro*. Hence, the main objective of the study was to decipher the roles of LINC00488-miR-376a-3p-PON2 pathways in thyroid cancer, thereby providing a deep understanding of the LINC00488’s function in thyroid cancer to develop it as a promising diagnostic and therapeutic target for this disease.

## Materials and methods

### Cell culture and treatment

Human normal thyroid cell line Nthy-ori3-1 (derived from human thyroid follicular epithelial normal cells) and human thyroid cancer cell lines (BCPAP, BHP5-16, TPC-1 and CGTH-W3) were purchased from Chinese Academy of Science (Shanghai, China). Cells were cultured in Roswell Park Memorial Institute (RPMI, KeyGEN, Nanjing, China) 1640 complete medium supplemented with 10% fetal bovine serum (FBS), 100 U/ml penicillin and 100 μg/ml streptomycin in an incubator at 37°C with 5% CO_2_ and saturated humidity. The CO_2_ cell incubator purchased from Forma Scientific U.K. and FACS Calibri flow cytometer purchased from BD Biosciences (U.S.A.). ABI7300 fluorescence quantitative PCR instrument was purchased from Applied Biosystems Inc.

### Cell counting kit-8 assay

Cells (1 × 10^4^ cells/well) were maintained in 96-well plates. For cell viability assessment, transfected cells were incubated for predetermined times (24, 48 and 72 h), respectively. After treatment, 10 μl of cell counting kit-8 (CCK-8) reagent (Beyotime, Shanghai, China) was added. The absorbance was measured at 450 nm (OD) with a microplate reader (BioTek Instruments Inc., Winooski, VT, U.S.A.). The experiment was repeated three times.

### EdU assay

When cell confluence reached approximately 80%, Cell-Light™ EdU fluorescence microscope detection kit (Keygen, Nanjing, China) was employed for BCPAP and TPC-1 cell proliferation detection in accordance with the manufacturer’s instructions. The cells were exposed to 50 μM EdU (5-ethynyl-2′-deoxyuridine; 100 μl/well, Syngene, Nanjing, China) for 2 h, fixed with PBS containing 4% paraformaldehyde (100 μl/well, Syngene, Nanjing, China) at room temperature for 15 min and incubated with 2 mg/ml glycine for 10 min. The cells were permeabilized with PBS comprising 0.5% Triton X-100 (100 μl/well) (Keygen, Nanjing, China) and stained with 100 μl of 1× Apollo dye liquor (Keygen, Nanjing, China) at room temperature for 30 min under conditions void of light. Incubation was continued following the addition of 100 μl of 4′,6-diamidino-2-phenylindole (DAPI) (Beyotime, Shanghai, China) staining solution at room temperature for 10–30 min in dark. After treating with DAPI, microscopic observation was performed under the guidance of a fluorescence microscope (Leica DM16000B, Germany). At least three fields were then selected from each well.

### Flow cytometry

Cell cycle assay was performed on BCPAP cells and TPC-1 cells. Following the 48-h incubation, approximately 1 × 10^6^ cells were seeded in cell culture flasks. After removing of the medium, the substance loaded medium was added, and the flasks were incubated for approximately 24–48 h. The living cells were collected by centrifugation (1500 rpm, 5 min) and washed twice with PBS. After careful fixation with ice-cold ethanol (70%, −20°C, 12 h), the cells were centrifuged (1500 rpm, 5 min) again, washed twice with PBS. Then, 500 μl Reagent A (1 mg/ml, 30 min, Syngene, Nanjing, China) was added in the dark at room temperature. Flow cytometry was used for detection. Data were plotted and analyzed by using FCS software (De Novo Software, Los Angeles, CA). The experiment was repeated three times.

Cell apoptosis was determined using Annexin V-FITC/PI apoptosis kit (Keygen, Nanjing, China) according to manufacturer’s instructions. After BCPAP cells and TPC-1 cells were incubated for 72 h, the cell suspension was prepared using 0.125% trypsin, centrifuged at 1500 rpm for 5 min, and then rinsed with ice-cold PBS. Cells were then resuspended in binding buffer (10 mM HEPES, pH 7.4, 140 mM NaCl and 2.5 mM CaCl_2_, KeyGEN, Nanjing, China) at a concentration of 1 × 10^6^ cells/ml. Subsequently, the cells were stained with Annexin V-FITC and propidium iodide (PI) for 20 min in the dark and analyzed by a flow cytometer. The experiments were repeated three times.

### Scratch assay

When cells reached 90% confluence, a single wound was created and phase-contrast images were digitally photographed immediately and 48 h after incubation. The original opening distances of the wound were set as 100%. The opening distances after 48 h were measured from three areas randomly selected per well, and the distances in three wells of each group were quantified and normalized by the original opening distance. The experiment was performed three times in triplicate, and the percentage of the migration rate was calculated by measuring the length of cell migration and expressed as a percentage compared with the control group. Migration rates = (treatment group cell migration distance/control group migration distance) × 100%.

### Transwell chamber assay

An 8-μm pore size transwell chamber without matrigel (Keygen, Nanjing, China) was used for transwell migration assay, and an 8-μm pore size transwell chamber with Matrigel (Keygen, Nanjing, China) was used for transwell invasion assay. Cells were digested and counted. A total of 1 × 10^6^ cells in 100 μl medium supplemented without FBS were plated in the upper chamber and 500 μl medium supplemented with 10% FBS was covered on the bottom chambers as chemoattractant. After 24-h incubation in a humidified incubator, non-migratory cells on the upper membrane surface were carefully removed, and those on the bottom surface were fixed with 4% polyoxymethylene (Sigma, MO, U.S.A.) and stained with 0.1% Crystal Violet (Sigma, MO, U.S.A.) for 15 min. Cells were counted by photographing five random fields under a microscope (BX53, Olympus, Tokyo, Japan) at 400× magnification and images were record.

### Western blot assay

Total protein was extracted using a RIPA kit (Keygen, Nanjing, China). The cells were lysed by protein lysis (60% RIPA + 39% sodium dodecyl sulfate (SDS) + 1% protease inhibitor) on ice for 30 min and then centrifuged. The protein concentration of the supernatant was determined using a bicinchoninic acid (BCA) kit (Beyotime, Shanghai, China). After that, the cell lysates were separated by SDS/PAGE (300 V, 30 min) and transferred on to a nitrocellulose membrane by means of wet transfer. Membrane blockade was conducted using 5% BSA for 1 h at room temperature and incubated with primary antibodies (Abcam, Cambridge, MA, U.K.): rabbit antibodies to CyclinD1 (ab226977), p21 (ab109520), Bax (ab182733), Bcl-2 (ab185002), Cleaved-caspase-3 (ab49822), Cleaved-caspase-9 (ab2324), matrix metalloproteinase (MMP) 2 (MMP-2; ab97779), MMP-9 (ab38898), PON2 (ab183710) and β-actin (ab8227) overnight at 4°C. The membranes were then incubated with the horseradish peroxidase (HRP)-conjugated goat anti-rabbit secondary antibody to IgG (Abcam, Cambridge, MA, U.K.). The results were visualized with an exposure machine, with β-actin regarded as an internal control. The film was scanned, the gray value was measured using the Wes automatic protein blot quantification analysis system, after which the relative ratio was calculated and subsequently compared with the internal reference. The experiment was repeated three times in each group.

### RNA isolation and quantification

Total RNA was extracted with TRIzol reagent (Thermo Fisher, U.S.A.) from treated and untreated cells as recommended by the manufacturer and purified with RNeasy Mini Kit (Qiagen, Maryland). The resulting cDNA samples were amplified by real-time PCR in the presence of SYBR Green Real time PCR Super Mix (Invitrogen, U.S.A.) in the ABI PRISM® 7500 Sequence Detection System (Applied Biosystems, U.S.A.). The amplification reaction mixture (25 μl) contained cDNAs, forward primers, reverse primers, and SYBR Green Real Time PCR Master Mix. Samples were analyzed in triplicate. PCR consisted of 35 cycles of 95°C for 10 s and then 60°C for 20 s, 72°C for 30 s. The primer set for each gene is listed below.

LINC00488, forward 5′-CAATACTGACCACATCCACGTC-3′; reverse 5′-GGGTCTGGCTCACTGTCTTTA-3′.
miR-376a-3p, forward 5′-CCCAGGAGGACTGAAGCAACAA-3′; reverse 5′-GCTATCTCAGGGCTTGTTGCTTC-3′.
PON2, forward 5′-ATATCTCTAGACCGCGGGGA-3′; reverse 5′-GGGTGTCGGAATAGACTCTG-3′.
U6, forward 5′-CTCGCTTCGGCAGCACA-3′; reverse 5′-AACGCTTCACGAATTTGCGT-3′;
GADPH, forward 5′-AGTUAGGCTGGGGCTCATTG-3′; reverse 5′-AGGGGCCATCCACAGTCTTC -3′.

U6 and GADPH were used as an internal control. PCR products were electrophoresed on a 1.5% agarose gel. The fold change in gene expression was calculated using 2^−ΔΔ*C*_T_^ method after normalizing to the expression level of U6 and GADPH.

### Dual-luciferase reporter gene assay

Bioinformatics prediction website was used to ascertain as to whether binding sites existed between LINC00488 and miR-376a-3p as well as between miR-376a-3p and 3′-untranslated region (3′-UTR) of PON2. Next, pmirGLO dual-luciferase miRNA target expression vector (Keygen, Nanjing, China) was performed to construct wildtype-LINC00488 (Wt-LINC00488) and mutant type-LINC00488 (Mut-LINC00488) vectors. The binding site between LINC00488 and miR-376a-3p was determined by means of dual-luciferase reporter gene assay. A full length of LIN00488 gene was inserted between the two enzyme sites, Xho I and Xba I. The PCR products were detached by Xho I and Xba I and subcloned into the psiCHECK-2 vector. The cells were seed into a six-well plate with 1 × 10^6^ cells per well and transfected in accordance with the aforementioned method. The successfully transfected cells were collected after a 48-h culture period. The effects of miR-376a-3p on luciferase activity of 3′-UTR of PON2 were detected based on the instructions provided by the dual-luciferase detection kit (Keygen, Nanjing, China). Glomax20/20 luminometer (Yuanpinghao, Beijing, China) was utilized for fluorescence intensity determination. The experiment was repeated three times.

### Immunocytochemistry

BCPAP and TPC-1 cells were fixed in 4% paraformaldehyde for 15 min at room temperature. The fixed cells were blocked with 5% normal goat serum for 1 h and were incubated with a diluted solution of the primary antibody (1:100, ab71333, Abcam, MA, U.S.A.) at 4°C overnight. Cells were then washed in PBS for three times and incubated for 1 h with secondary biotin-labeled goat anti-rabbit antibody to immunoglobulin G (1:1000, ab6721, Abcam, MA, U.S.A.). Nuclei were counterstained with DAPI (Beyotime Biotechnology, Shanghai, China). Preparations were then observed with a fluorescent microscope (Leica DM16000B, Germany) and images were recorded.

### Statistical analysis

All experiment data were analyzed using the Statistic Package for Social Science (SPSS) 19.0 statistical software (IBM Corp., Armonk, NY, U.S.A.). The experiments were repeated three times. Measurement data were expressed as mean ± standard deviation (SD). The differences between two groups were assessed using the Student’s *t* test. The differences among multiple groups were analyzed by one-way ANOVA followed by the Tukey’s post hoc test. The differences on cell proliferation at different time points were analyzed by repeated-measurement ANOVA. *P*<0.05 was considered to be statistically significant.

## Results

### Knockdown of LINC00488 inhibits proliferation and promotes apoptosis of thyroid cancer cells (BCPAP and TPC-1)

**Figure 1:**
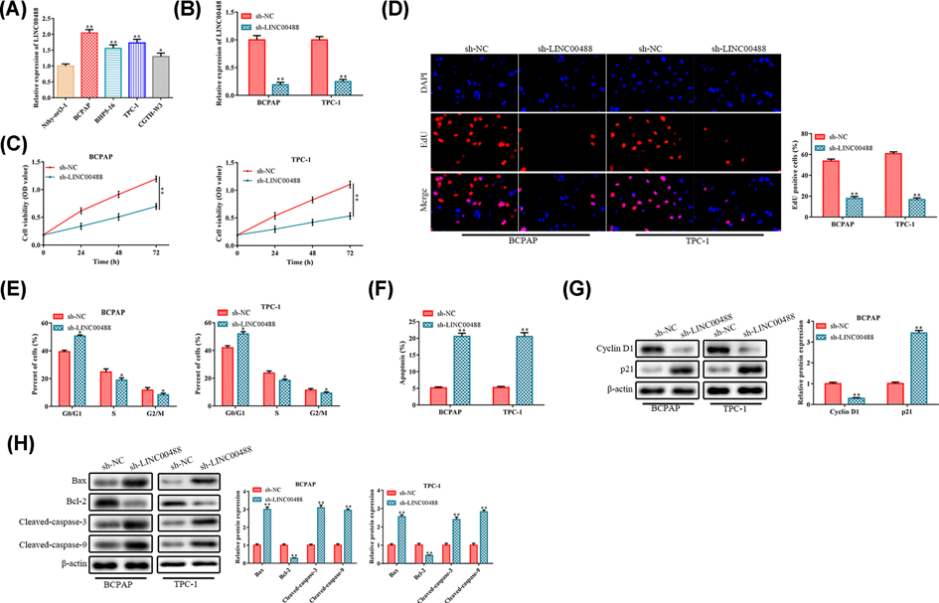
Knockdown of LINC00488 inhibits proliferation and promotes apoptosis of thyroid cancer cells BCPAP and TPC-1 cells were transfected with either LINC00488 shRNAs or negative control (NC) shRNAs. (**A**) The expression level of lncRNA LINC00488 in normal thyroid cells (Nthy-ori3-1) and thyroid cancer cell lines (BCPAP, BHP5-16, TPC-1 and CGTH-W3) were determined by RT-qPCR. *P<0.05, **P<0.01 *vs.* the Nthy-ori3-1 cell line. (**B**) RT-qPCR analysis of the cell transfection. (**C**) CCK-8 assay for the cell viability. (**D**) EdU assay for the cell proliferation. (**E**) Flow cytometry for the cell cycle distribution. (**F**) Flow cytometry for the cell apoptosis. (**G**) Western blot analysis for the protein expression levels of proliferation-related proteins. (**H**) Western blot analysis for the protein expression levels of apoptosis-related proteins. *P<0.05, **P<0.01 *vs.* the sh-NC group. The data were presented as mean ± SD and the experiments were repeated three times.

RT-qPCR analysis was used to examine the expression level of LINC00488 in human normal thyroid cell line Nthy-ori3-1 and human thyroid cancer cell lines (BCPAP, BHP5-16, TPC-1 and CGTH-W3). Compared with Nthy-ori3-1 cell line, the LINC00488 expression in the human thyroid cancer cell lines was markedly increased, among which the BCPAP cell line and TPC-1 cell line exhibited the highest LINC00488 expression (Figure 1A). Therefore, the thyroid cancer cell BCPAP and TPC-1 were selected for the subsequent experiments.

In order to evaluate the effect of LINC00488 on cell proliferation and apoptosis, the LINC00488 shRNA was transfected into the cells (BCPAP and TPC-1). The RT-qPCR analysis indicated that the expression level of LINC00488 was significantly decreased in the sh-LINC00488-transfected cells (Figure 1B). CCK-8 and EdU incorporation assays indicated that LINC00488 knockdown significantly inhibited proliferation of BCPAP and TPC-1 cell lines (Figure 1C,D). In addition, flow cytometry analysis found that LINC00488 knockdown made more cells arrested at the G_0_/G_1_ stage and fewer cells at G_2_/M stage and promoted the apoptosis both in BCPAP and TPC-1 cell lines (Figure 1E,F). At the molecular level, knockdown of LINC00488 significantly decreased the protein expression of Cyclin D1 and Bcl-2, while increased the p21, Bax, Cleaved-Caspase-3 and Cleaved-Caspase-9 (Figure 1G,H). Based on the aforementioned results, we concluded that knockdown of LINC00488 could inhibit the proliferation and promote apoptosis of thyroid cancer cells.

### Knockdown of LINC00488 represses migration and invasion of thyroid cancer cells

**Figure 2:**
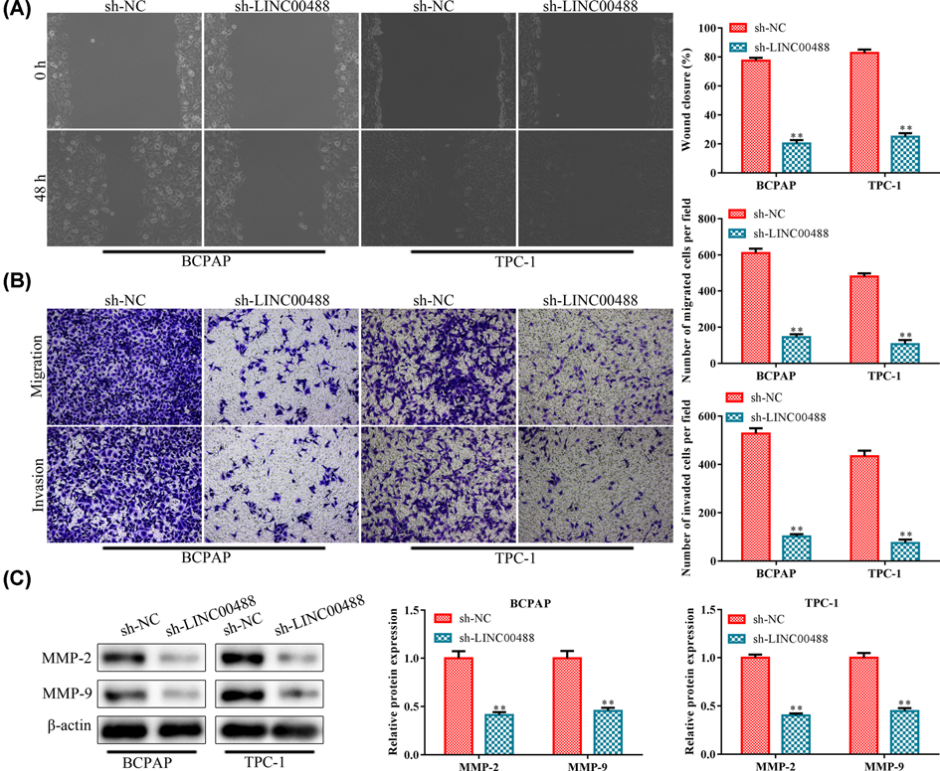
Knockdown of LINC00488 represses migration and invasion of thyroid cancer cells Cells were treated similarly as in Figure 1. (**A**) Cell capacity of migration (scratch test). (**B**) Transwell chamber assay for cell migration and invasion. (**C**) Western blot analysis of the expression level of migration related proteins (MMP-2 and MMP-9). **P<0.01 *vs.* the sh-NC group. The data were presented as mean ± SD and the experiments were repeated three times.

To evaluate the effects of LINC00488 knockdown on the cell migration and invasion in BCPAP and TPC-1 cells, wound scratch and transwell chamber assays were performed. The results of the wound scratch assay showed that the wound closure of the distance in the sh-LINC00488 group was significantly decreased compared with that in the sh-NC group (Figure 2A). Meanwhile, transwell chamber assay indicated that the number of migratory cells and invasive cells in the sh-LINC00488 group was markedly reduced, compared with the sh-NC group (Figure 2B). Given the critical function of MMP-2 and MMP-9 in tumor cell migration and invasion, we investigated the effect of sh-LINC00488 on the protein expression levels of MMP-2 and MMP-9. Western blot analysis showed that both of these two proteins were decreased in the sh-LINC00488-treated BPCAP and TPC-1 cells (Figure 2C). All the above results demonstrated that LINC00488 knockdown could repress migration and invasion of thyroid cancer cells (BCPAP and TPC-1), which indicated that LINC00488 was a potential activator for the migration and invasion of thyroid cancer cells.

### LINC00488 directly binds to miR-376a-3p and down-regulates the expression of miR-376a-3p

**Figure 3:**
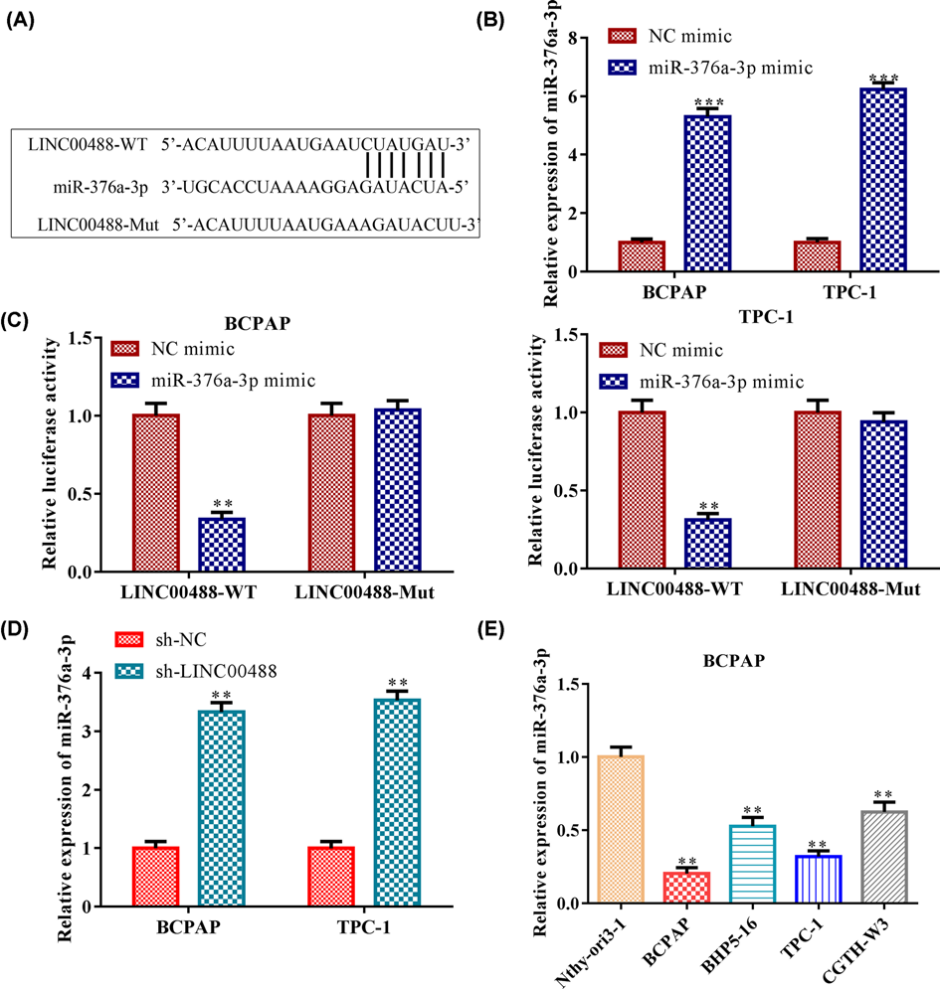
LINC00488 directly binds to miR-376a-3p and down-regulates the expression of miR-376a-3p (**A**) The predicted LINC00488 binding sites in the region of miR-376a-3p and the corresponding mutant sequence were shown. (**B**) The expression level of miR-376a-3p both in BCPAP and TPC-1 cells after transfected with NC mimic and miR-376a-3p mimic, respectively. ***P<0.001 *vs.* the NC mimic group. (**C**) Relative values of luciferase signal. **P<0.01 *vs.* the NC mimic group. (**D**) The expression level of miR-376a-3p both in BCPAP and TPC-1 cells after transfected with sh-NC and LINC00488 shRNAs, respectively. **P<0.01 *vs.* the sh-NC group. (**E**) The expression level of miR-376a-3p in normal thyroid cell and thyroid cancer cell lines. **P<0.01 *vs.* the Nthy-ori3-1 cell line. The data were presented as mean ± SD and the experiments were repeated three times.

LncRNAs have been demonstrated to serve as competing endogenous RNAs (ceRNAs), which sponge microRNAs (miRNAs) to regulate the expression of miRNAs. To find out the specific miRNA that was regulated by LINC00488, we performed bioinformatics analysis and dual-luciferase reporter gene assay. The results of bioinformatics analysis DIANA suggested that miR-376a-3p, which contained the putative binding sites, was the target of LINC00488 (Figure 3A). First, we validated that the miR-376a-3p expression was significantly increased in the BCPAP and TPC-1 cells after treating miR-376a-3p mimic compared with the NC mimic group (Figure 3B). Furthermore, dual-luciferase reporter gene assay was employed to further ascertain whether LINC00488 could competitively sponge miR-376a-3p in the BCPAP and TPC-1 cells. The results revealed that the relative luciferase activity of LINC00488-Wt was obviously decreased by miR-376a-3p mimic, whereas no similar reduction was observed in the luciferase activity of LINC00488-Mut (Figure 3C). Consistently, we assessed the level of miR-376a-3p in thyroid cancer cells (BPCAP and TPC-1) transfected with sh-LINC00488. We found that the level of miR-376a-3p was markedly increased in response to LINC00488 knockdown (Figure 3D). In addition, the expression level of miR-376a-3p in human normal thyroid cell line Nthy-ori3-1 and human thyroid cancer cell lines (BCPAP, BHP5-16, TPC-1 and CGTH-W3) was detected. Compared with Nthy-ori3-1 cell line, the miR-376a-3p expression in the human thyroid cancer cell lines was markedly decreased (Figure 3E). Taken together, LINC00488 could directly bind to the miR-376a-3p and the knockdown of LINC0048 could down-regulation the expression of miR-376a-3p.

### MiR-376a-3p targets PON2 and causes post-transcriptional suppression

**Figure 4:**
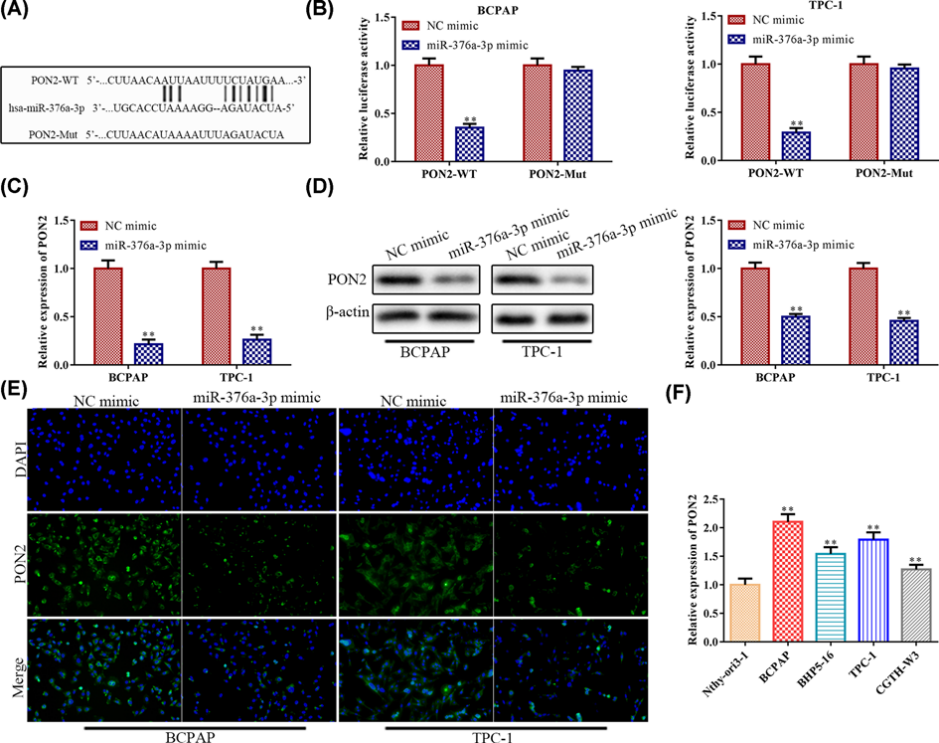
MiR-376a-3p targets PON2 and causes post-transcriptional suppression (**A**) The predicted miR-376a-3p binding sites in the region of PON2 and the corresponding mutant sequence were shown. (**B**) Relative values of luciferase signal. (**C**) The expression level of PON2 both in BCPAP and TPC-1 cells was determined by RT-qPCR after transfected with NC mimic and miR-376a-3p mimic, respectively. (**D**) Western blot analysis of the expression level of PON2 protein. **P<0.01 *vs.* the NC mimic group. (**E**) Immunocytochemistry assay. (**F**) The expression level of PON2 in normal thyroid cell and thyroid cancer cell lines. **P<0.01 *vs.* the Nthy-ori3-1 cell line. The data were presented as mean ± SD and the experiments were repeated three times.

In order to explore the potential anti-tumor mechanism of miR-376a-3p, bioinformatics analysis of TargetScan was used to predict potential target of miR-376a-3p. Among these targets, PON2 was considered to be a potential target of miR-376a-3p in thyroid cancer since its critical role in the regulation of tumor progression [20]. As shown in Figure 4A, the 3′-UTR of the PON2 contained a putative binding site of miR-376a-3p. The regulatory effect of miR-376a-3p and PON2 was further validated by the dual-luciferase reporter gene assay. The results showed that miR-376a-3p mimic was able to inhibit the luciferase activity of PON-Wt compared with mimic-NC. However, no significance changes were observed in the luciferase activity of PON2-Mut (Figure 4B), indicating that PON2 was a direct target of miR-376a-3p in BCPAP and TPC-1 cells. Then, we detected the expression of PON2 in BCPAP and TPC-1 cells through RT-qPCR, Western blot assay and immunocytochemistry. We found that the expression level of PON2 markedly decreased in cells transfected with miR-376a-3p mimic (Figure 4C–E). In addition, we validate the mRNA expression levels of PON2 in human normal thyroid cell line Nthy-ori3-1 and human thyroid cancer cell lines (BCPAP, BHP5-16, TPC-1 and CGTH-W3). The results indicated that the expression level of PON2 was increased in cancer cells (Figure 4F), indicating a negative correlation between miR-376a-3p and PON2 expression. Therefore, PON2 was a target gene of miR-376a-3p and was negatively regulated by miR-376a-3p.

### MiR-376a-3p/PON2 mediates the inhibitory effects of sh-LINC00488 on the thyroid cancer cell progression

**Figure 5:**
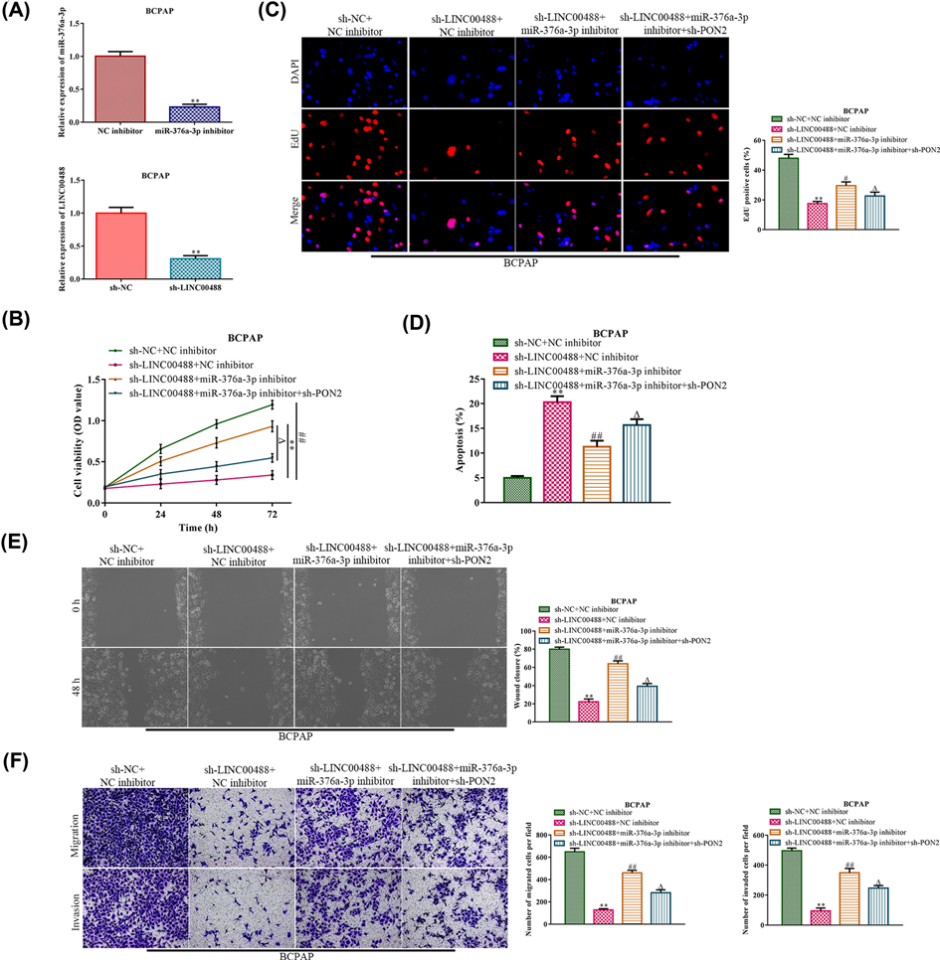
MiR-376a-3p/PON2 mediates the inhibitory effects of sh-LINC00488 on the thyroid cancer cell progression Either miR-376a-3p inhibitor or PON2 shRNAs were transfected into the LINC00488 knockdown BCPAP cells. (**A**) RT-qPCR was performed to evaluate the efficiency of miR-376a-3p inhibitor and LINC00488 knockdown. **P<0.01 *vs.* the NC inhibitor group and the sh-NC group, respectively. (**B**) CCK-8 assay for the cell viability. (**C**) EdU assay for the cell proliferation. (**D**) Flow cytometry for the cell apoptosis. (**E**) Cell capacity of migration (scratch test). (**F**) Transwell chamber assay for cell migration and invasion. **P<0.01 *vs.* the sh-NC + NC inhibitor group, ^#^*P*<0.05, ^##^*P*<0.01 *vs.* the sh-LINC00488 + NC inhibitor group, and ^Δ^*P*<0.05 *vs.* the sh-LINC00488 + miR-376a-3p inhibitor group. The data were presented as mean ± SD and the experiments were repeated three times.

To test whether miR-376a-3p/PON2 axis was involved in LINC00488-promoted thyroid cancer progression, miR-376a-3p inhibitor and PON2 shRNAs were transfected into BCPAP cells in the presence with LINC00488 shRNAs. The efficiency of miR-376a-3p inhibition and LINC00488 knockdown in BCPAP were present in Figure 5A. CCK-8 and EdU incorporation assays indicated that miR-376a-3p inhibitor induced BCPAP proliferation in the BCPAP cells with lncRNA LINC00488 knockdown, while these induction effects were partially antagonized by knockdown of PON2 (Figure 5B,C). In contrast, knockdown of PON2 recapitulated the miR-376a-3p inhibitor-induced suppression of cell apoptosis in the LINC00488-deficient BCPAP cells (Figure 5D). Coinciding with these results, the cell migration and invasion analyses revealed the similar changes as in the proliferation analysis (Figure 5E,F). Taken together, lncRNA LINC00488 activated thyroid cancer cell progression by targeting the miR-376a-3p/PON2 axis.

## Discussion

Thyroid cancer is the most common malignant tumor in endocrine system and its incidence has been steadily increasing in the world [21,22]. Therefore, great efforts should be made to comprehensively understand the development of the thyroid cancer. Previously it has been reported that lncRNAs in thyroid cancer have become potential prognostic indicators for therapeutic intervention [23–25]. The main reason is that lncRNAs regulates gene expression at both transcriptional and post-transcriptional levels in all fundamental cellular processes, including proliferation, differentiation, immunity, altered metabolism and signaling, as well as cancer progression [26,27]. The clinical significance and biological function of lncRNA LINC00488 in thyroid cancer is still elusive. In the current study, we characterized the expression pattern and molecular mechanism of LINC00488 in thyroid cancer. Our findings revealed that LINC00488 was highly expressed in thyroid cancer cell lines and knockdown of LINC00488 inhibited the cell proliferation, migration and invasion and promoted the cell apoptosis in thyroid cancer cell lines. Mechanistically, LINC00488 served as oncogenic gene in thyroid cancer progression through regulation of miR-376a-3p/PON2 axis.

The interaction between miRNAs and lncRNAs within cells has been shown to be one representative regulation pattern of miRNAs and should be discussed due to their large number. Studies have showed that the expression of lncRNAs can regulate the activities of miRNAs [28]. MiRNAs belong to a class of endogenous, small non-coding RNAs containing approximately 22 nucleotides which are involved in regulation of downstream target genes expression at the post-transcriptional level [29]. The researches revealed that aberrant expression of miRNAs was related to tumorigenesis and metastasis of cancers [30–33].

The underlying mechanism by which lncRNAs promote the proliferation and inhibit the apoptosis of cancer involve transcription or post-transcription, epigenetic modification and miRNA processing [13,32]. It is now increasingly acknowledged that lncRNAs regulate development and progression via sponging an array of downstream miRNAs. Indeed, LINC00488 has been reported to sponge a mass of miRNAs in a variety of cancers. For example, in hepatocellular carcinoma, LINC00488 acts as a ceRNA, which could competitively sponge miR-330-5p to regulate TLN1, thus affecting the cell growth and angiogenesis in HCC [12]. Of note, previous study has demonstrated that the miR-376a-3p expression is related with the tumor development of various malignancies, including thyroid cancer [32]. For example, lncRNA TTN-AS1 could sponge miR-376a-3p to promote colorectal cancer development by regulating KLF5 [34]. In addition, miRNA-376a-3p overexpression inhibited the progression of coronary artery disease through regulating NRIP1 [35]. Therefore, we selected it as the target of LINC00488. However, the relationship between LINC00488 and miR-376a-3p was unclear. In our study, we performed the bioinformatics analysis and the dual luciferase report assay to validate the predication. Meanwhile, the expression level of miR-376a-3p was down-regulation in thyroid cancer cells and was negatively correlated with the expression of LINC00488. Besides, by using TargetScan platform, dual luciferase report gene assay, RT-qPCR and Western blot, we also identified the PON2 was a target gene of miR-376a-3p, while overexpression of miR-376a-3p could decrease the expression of PON2. Last but not least, rescue experiments demonstrated that the essential role of miR-376a-3p-PON2 axis in mediating the effects of LINC00488 on the thyroid cancer cell progression. Collectively, our findings indicated that LINC00488 might enhance PON2 expression by sequestering the miR-376a-3p in thyroid cancer.

In summary, our study revealed that lncRNAs LINC00488 was highly expressed in thyroid cancer cell lines. Knockdown of LINC00488 decreased proliferation, migration and invasion, while activated apoptosis of thyroid cancer cells. The underlying mechanism of LINC00488 was that LINC00488 might enhance PON2 expression by sponging the miR-376a-3p expression. Therefore, the LINC00488-miR-376a-3p-PON2 axis may serve as novel biomarkers or potential targets for the treatment of thyroid cancer.

## Data Availability

All data generated or analysed during this study are included in this published article.

## Abbreviations

ANOVA: analysis of variance
ATC: anaplastic thyroid cancer
BCA: bicinchoninic acid
CCK-8: cell counting kit-8
DAPI: 4′,6-diamidino-2-phenylindole
EdU: 5-ethynyl-2′-deoxyuridine
FBS: fetal bovine serum
FTC: follicular thyroid cancer
GADPH: glyceraldehyde-3-phosphate dehydrogenase
lncRNA: long non-coding RNA
miRNA: microRNA
MMP: matrix metalloproteinase
OD: optical density
PI: propidium iodide
PON2: Paraoxonase-2
PTC: papillary thyroid cancer
RT-qPCR: real time quantitative polymerase chain reaction
SD: standard deviation
SDS: sodium dodecyl sulfate
3′-UTR: 3′-untranslated region
